# Research on vitamin D metabolic regulation in the pathogenesis of related diseases

**DOI:** 10.3389/fendo.2026.1762302

**Published:** 2026-03-18

**Authors:** Yumeng Ji, Ruru Bi, Lin Wang

**Affiliations:** Center of Clinical Laboratory, The Fourth Affiliated Hospital of Soochow University, Suzhou Dushu Lake Hospital, Suzhou, China

**Keywords:** vitamin D metabolism, chronic kidney disease, mineral and bone disorder, CYP27B1, CYP24A1, precision medicine

## Abstract

Vitamin D is a group of fat-soluble vitamins that plays critical roles in calcium-phosphate homeostasis, bone health, and immune regulation. The metabolic pathway of vitamin D involves two key enzymatic steps: hepatic 25-hydroxylation to produce 25-hydroxyvitamin D [25(OH)D] and renal 1α-hydroxylation to generate the biologically active form 1,25-dihydroxyvitamin D [1,25(OH)_2_D]. 25(OH)D serves as the gold standard biomarker for assessing vitamin D status due to its high circulating concentration, long half-life, and stable levels. This metabolic process is precisely regulated by parathyroid hormone (PTH), fibroblast growth factor 23 (FGF23), and negative feedback mechanisms mediated by the vitamin D receptor (VDR). Recent studies have demonstrated that CYP27B1, the gene encoding 1α-hydroxylase, is also widely expressed in extrarenal tissues including prostate, breast, placenta, and macrophages, suggesting important roles for vitamin D in local paracrine and autocrine regulation. Dysregulation of vitamin D metabolism is closely associated with the pathogenesis of various diseases, including vitamin D-dependent rickets type 1A (VDDR-1A), chronic kidney disease-mineral and bone disorder (CKD-MBD), tumor-induced osteomalacia (TIO), granulomatous diseases, and CYP24A1 deficiency-related hypercalcemia. Notably, 24,25-dihydroxyvitamin D [24,25(OH)_2_D], traditionally considered an inactive metabolic end-product, may possess independent biological functions, and its ratio to 1,25(OH)_2_D can serve as a novel biomarker for assessing vitamin D metabolic status. This review systematically examines the metabolic pathways and regulatory mechanisms of vitamin D, elucidates their associations with disease pathogenesis, and provides theoretical foundation for personalized clinical diagnosis and precision therapy. Future research should establish standardized multi-parameter detection protocols to advance the clinical application of vitamin D metabolomics in precision medicine.

## 1 Introduction

Vitamin D is a class of fat-soluble vitamins, including ordinary vitamin D [plant-derived ergocalciferol (vitamin D_2_) and animal-derived cholecalciferol (vitamin D_3_)] as well as activated vitamin D [25-hydroxyvitamin D_3_ (25(OH)D_3_), 1α-hydroxyvitamin D_2_ (1α-(OH)D_2_), 1α-hydroxyvitamin D_3_ (1α-(OH)D_3_), 1,25-dihydroxyvitamin D_3_ (1,25(OH)_2_D_3_), and 19-nor-1,25-dihydroxyvitamin D_2_] (1).In the human body, 80%-90% of vitamin D originates from 7-dehydrocholesterol in subcutaneous tissue, which is synthesized into vitamin D_3_ through skin exposure to ultraviolet B radiation from sunlight; the remaining 10%-20% of vitamin D comes from ordinary vitamin D in food (vitamin D_2_ and vitamin D_3_) (1). Regarding exogenous intake, vitamin D_2_ is mainly derived from plants and fungi, obtained through food and supplements; vitamin D_3_ can be acquired through animal-based foods (such as fish, egg yolk, and dairy products) as well as fortified foods, and can also be supplemented in the form of dietary supplements.Vitamin D is converted to 25(OH)D by hepatic 25-hydroxylase in the body, and then converted by renal 1α-hydroxylase into the biologically active 1,25(OH)_2_D, or metabolized by 24-hydroxylase to 24,25(OH)_2_D for degradation (2). Recent studies have shown that vitamin D metabolism is crucial for maintaining calcium-phosphorus balance, skeletal health, and immune function, and is closely related to the pathogenesis of various common diseases, such as osteoporosis, cardiovascular disease, and diabetes, thus becoming an important clinical biomarker and therapeutic target (3). A recent individual participant data meta-analysis explicitly demonstrates that daily physiological dosing (400-2000 IU), rather than intermittent bolus administration, reduces cancer mortality by 12%, optimizing clinical protocols for precision dosing (4). This article reviews the metabolic processes and regulation of vitamin D, explores the associations with the pathogenesis of various related diseases, and aims to provide theoretical basis for individualized and precise clinical diagnosis and treatment. Large-scale cohort studies have confirmed the link between vitamin D deficiency and all-cause mortality. Emerging evidence suggests that maintaining serum 25(OH)D levels above 30 ng/mL could significantly reduce the disease burden for eight of the top ten global causes of death, providing a new targeted standard for population-level precision prevention (5). This review synthesizes current knowledge on vitamin D metabolism and regulation, examines its relationship with various disease pathogenesis, and aims to provide a theoretical foundation for personalized and precision clinical practice (6).

## 2 Vitamin D metabolic processes

**Figure 1:**
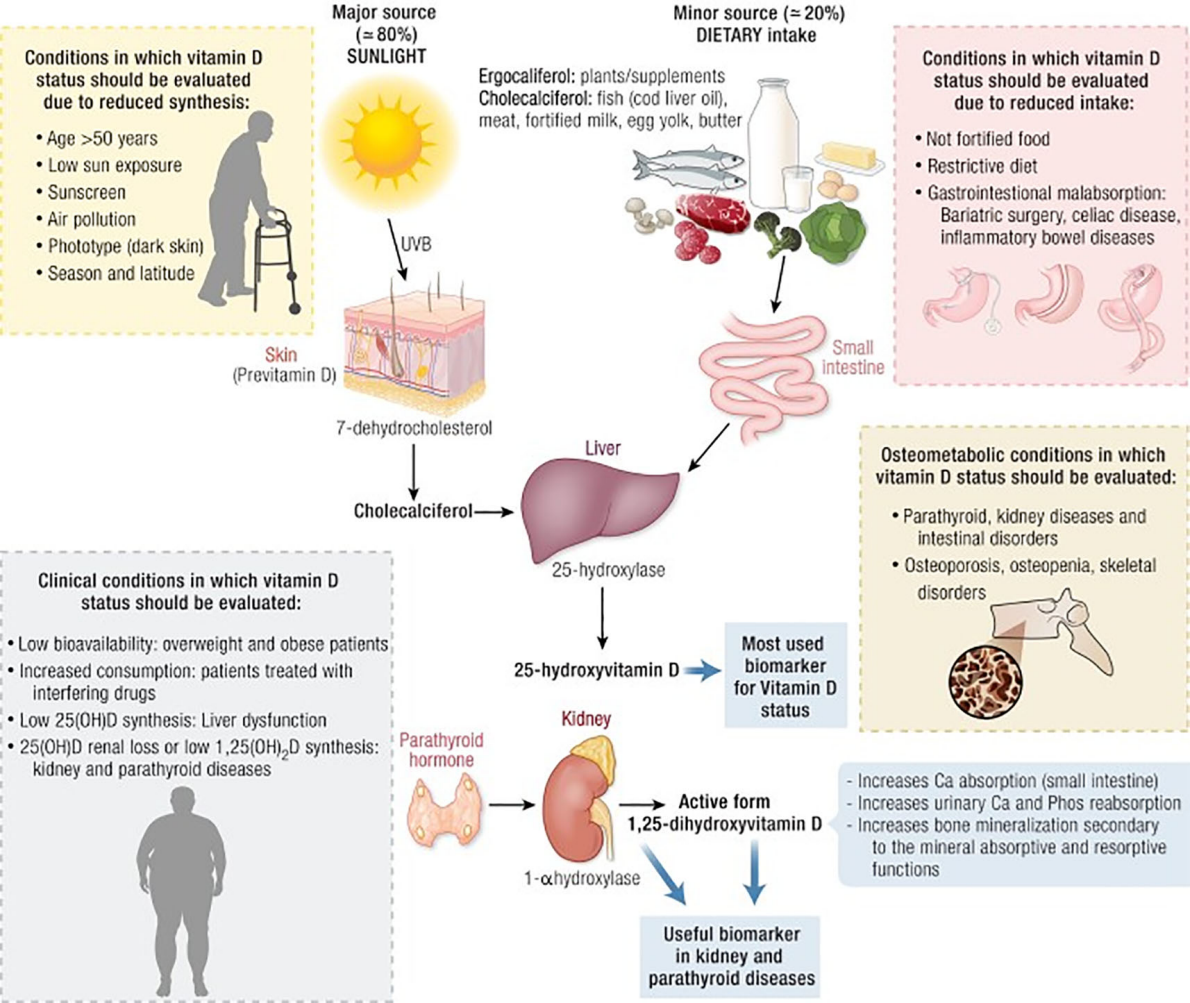
The figure shows Metabolic pathway of vitamin D in the body, with specific reference to conditions in which vitamin D should be evaluated (8).

Upon entering the bloodstream, vitamin D immediately binds with vitamin D binding protein (DBP) to form a stable transport complex. DBP is a multifunctional carrier protein produced by the liver, belonging to the albumin family, with a single binding site that has high affinity for all vitamin D metabolites. The vitamin D complex not only prevents rapid clearance of vitamin D from the blood but also regulates its bioavailability and maintains a stable reserve of vitamin D metabolites in circulation (7) (**Figure 1**).

### 2.1 Storage of vitamin D in the liver

The liver is the primary site for the initial hydroxylation of vitamin D, a process mainly catalyzed by the cytochrome P450 enzyme system (9). CYP2R1(Cytochrome P450 Family 2 Subfamily R Member 1) is recognized as the most important 25-hydroxylase, responsible for converting vitamin D_2_ and D_3_ into 25(OH)D_2_ and 25(OH)D_3_, respectively.CYP27A1(Cytochrome P450 Family 27 Subfamily A Member 1), as an auxiliary 25-hydroxylase, plays an important compensatory role when CYP2R1 function is impaired or insufficient.25(OH)D is the major circulating and storage form of vitamin D; due to its high blood concentration, long half-life (2–3 weeks), and stable levels, it accurately reflects the body’s vitamin D reserves, and thus has become the gold standard indicator for clinical assessment of vitamin D nutritional status (10). It is noteworthy that liver diseases such as non-alcoholic fatty liver disease and cirrhosis can significantly affect 25-hydroxylase activity, leading to abnormal vitamin D metabolism (11).

### 2.2 Activation and degradation of vitamin D in the kidney

In the metabolic pathway of vitamin D, the kidney is the key site for producing its biologically active form. The CYP27B1 (Cytochrome P450 Family 27 Subfamily B Member 1) gene expressed in renal proximal tubule cells encodes 1α-hydroxylase, which is responsible for hydroxylating the inactive circulating 25(OH)D into the fully biologically active 1,25(OH)_2_D, namely calcitriol (12). This hormone-like action is mainly achieved through the following mechanism: after 1,25(OH)_2_D binds to VDR within target cells (such as intestinal epithelial cells), it forms a heterodimer with retinoid X receptor (RXR); this complex then binds to the promoter region of specific genes, regulating the transcription of downstream target genes (13). One of its most essential physiological functions is to significantly promote intestinal absorption of calcium and phosphorus, providing adequate raw materials for bone mineralization (12). Meanwhile, it can also directly act on osteoblasts and indirectly affect osteoclasts, jointly maintaining the balance of bone metabolism.

The degradation of vitamin D is primarily mediated by CYP24A1 (Cytochrome P450 Family 24 Subfamily A Member 1) expressed in the kidney. CYP24A1 is the primary enzyme mediating vitamin D inactivation; however, its metabolic kinetics are significantly altered under specific pathological conditions. Recent metabolomic insights (Siwakoti et al., 2025) demonstrate that in the uremic environment, the catabolic rate of 1,25(OH)_2_D_3_ is markedly accelerated due to the aberrant activation of non-canonical degradation pathways, providing a deeper mechanistic explanation for the severe deficiency of active vitamin D in CKD patients (14). Ultimately, the metabolites are further oxidized into water-soluble products such as calcitroic acid, which are excreted from the body through the kidneys and biliary tract. This complete metabolic network not only ensures the timely activation of vitamin D biological activity but also ensures the prompt clearance of its metabolites, maintaining the dynamic balance of calcium-phosphorus metabolism in the body (15).

## 3 Regulation of vitamin D metabolism

### 3.1 Compensatory regulation by extrarenal organs

**Figure 2:**
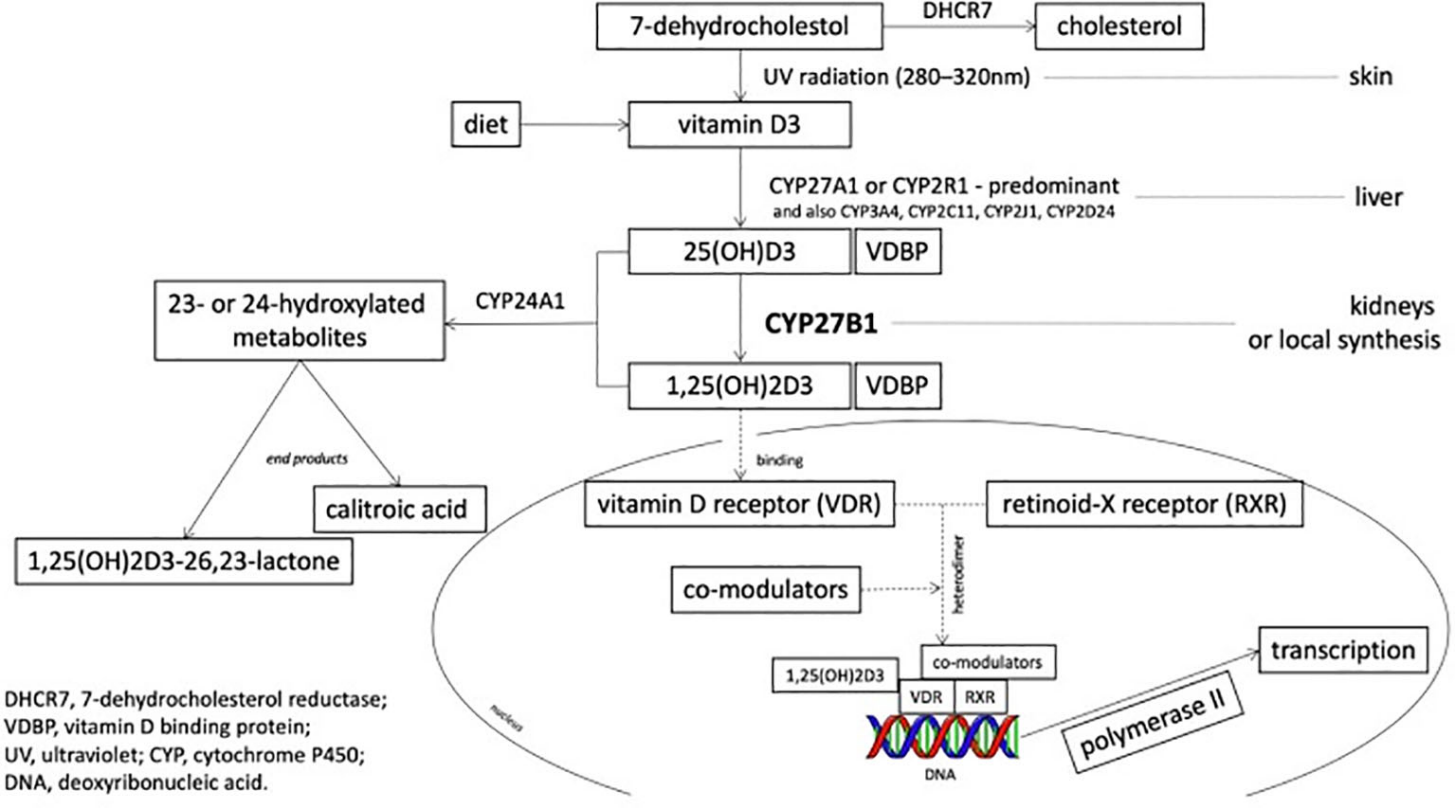
Schematic diagram of the CYP27B1 active site in the vitamin D metabolic pathway (17).

The expression and activity of CYP27B1 are positively regulated by PTH; when blood calcium levels decrease, PTH upregulates CYP27B1 gene transcription through the cAMP-PKA signaling pathway, promoting the synthesis of 1,25(OH)_2_D_3_ (13). As a nuclear hormone receptor ligand, 1,25(OH)_2_D_3_ regulates the transcriptional expression of hundreds of genes in target cells by binding to VDR, exerting multiple physiological functions such as regulating calcium-phosphorus homeostasis, bone mineralization, and immune modulation (16) (**Figure 2**).

In recent years, studies have shown that CYP27B1 is also widely expressed in tissues and organs outside the kidney, such as mammary glands, prostate, and macrophages, and may play an important compensatory role when renal function is abnormal (18). Therefore, this provides a new perspective on the mechanisms by which vitamin D functions in immune regulation, tumor prevention and treatment, and reproductive health (19).

#### 3.1.1 Prostate tissue: local vitamin D metabolism and tumor suppression

Prostate tissue is one of the important sites of extrarenal vitamin D metabolism; locally produced 1,25(OH)_2_D not only participates in the regulation of prostate cell proliferation but also plays an important protective role in the development and progression of prostate cancer (20). Downregulation of CYP27B1 in prostate cancer cells is linked to poor prognosis. A 2025 breakthrough study (Hendi et al., 2025) utilizing CRISPR technology identified that the SDR42E1 gene modulates vitamin D uptake, directly influencing tumor sensitivity to metabolic interventions and serving as a novel genetic marker for personalized precision therapy (21).

#### 3.1.2 Mammary tissue: CYP27B1 regulation of epithelial differentiation and breast cancer risk

CYP27B1 expression in mammary tissue significantly increases during pregnancy and lactation, and this change in expression pattern is closely related to the differentiation and functional maturation of mammary epithelial cells (22). The 1,25(OH)_2_D produced locally in mammary tissue during pregnancy not only participates in the regulation of calcium-phosphorus metabolism but also regulates the self-renewal and differentiation of mammary stem cells through activation of the VDR signaling pathway. Epidemiological studies have found that vitamin D deficiency during pregnancy is associated with increased breast cancer risk, which may be related to impaired local immune regulatory function caused by insufficient extrarenal CYP27B1 activity (23).

#### 3.1.3 Placenta: local 1,25(OH)_2_D production in maternal-fetal immune tolerance and fetal development

The placenta is one of the earliest tissues discovered to possess extrarenal 1α-hydroxylase activity, with CYP27B1 being expressed in placental syncytiotrophoblasts, cytotrophoblasts, stromal cells, and invasive trophoblast cells. At the maternal-fetal interface, CYP27B1 is also expressed in maternal decidual stromal cells and resident macrophages. The 1,25(OH)_2_D produced locally in the placenta plays a key role in maintaining immune tolerance at the maternal-fetal interface, regulating the depth of trophoblast invasion, and fetal skeletal development (24). Vitamin D deficiency during pregnancy not only affects placental CYP27B1 expression but may also lead to the occurrence of pregnancy complications such as preeclampsia and fetal growth restriction. Large prospective cohort studies have confirmed that vitamin D levels during pregnancy are positively correlated with neonatal birth weight and bone density, and can reduce the risk of gestational diabetes (25).

#### 3.1.4 Monocyte/macrophage system: CYP27B1-driven antimicrobial immunity and antiviral defense

CYP27B1 activity in the monocyte/macrophage system plays an important role in anti-infection immune responses (26). When the body encounters infection, activated macrophages increase local 1,25(OH)_2_D production by upregulating CYP27B1 expression; this local action not only promotes the expression of antimicrobial peptides such as cathelicidin and β-defensin but also maintains appropriate immune response intensity by regulating T cell differentiation. While epidemiological evidence links vitamin D deficiency to increased tuberculosis susceptibility, clinical mechanistic models suggest this is mediated through the impairment of macrophage CYP27B1-driven antimicrobial peptide production (27).

### 3.2 Interactions between regulatory factors and 1,25(OH)_2_D

#### 3.2.1 Regulatory effects of parathyroid hormone and fibroblast growth factor on 1,25(OH)_2_D

As the biologically active form of vitamin D, 1,25(OH)_2_D_3_ is primarily regulated by PTH in its synthesis; when serum calcium ion concentration decreases or 1,25(OH)_2_D_3_ levels are insufficient, the parathyroid gland increases PTH secretion, and PTH promotes the conversion of 25(OH)D_3_ to 1,25(OH)_2_D_3_ by activating renal CYP27B1 (28). Simultaneously, PTH inhibits CYP24A1 expression, preventing the catabolism of 1,25(OH)_2_D_3_, thereby maintaining its serum concentration.

Fibroblast growth factor 23 (FGF23), a bone-derived phosphate-regulating hormone, inhibits the synthesis of 1,25(OH)_2_D_3_ by downregulating the transcriptional expression of CYP27B1, while simultaneously upregulating CYP24A1 expression, promoting the metabolism of 1,25(OH)_2_D_3_ and 25(OH)D_3_ to the inactive 24,25(OH)_2_D_3_, and accelerating their clearance (29). More importantly, there is a negative feedback regulatory loop between 1,25(OH)_2_D_3_ and FGF23; 1,25(OH)_2_D_3_ can directly upregulate FGF23 gene expression in osteocytes, while increased FGF23 in turn inhibits the production of 1,25(OH)_2_D_3_ (30).

**Figure 3:**
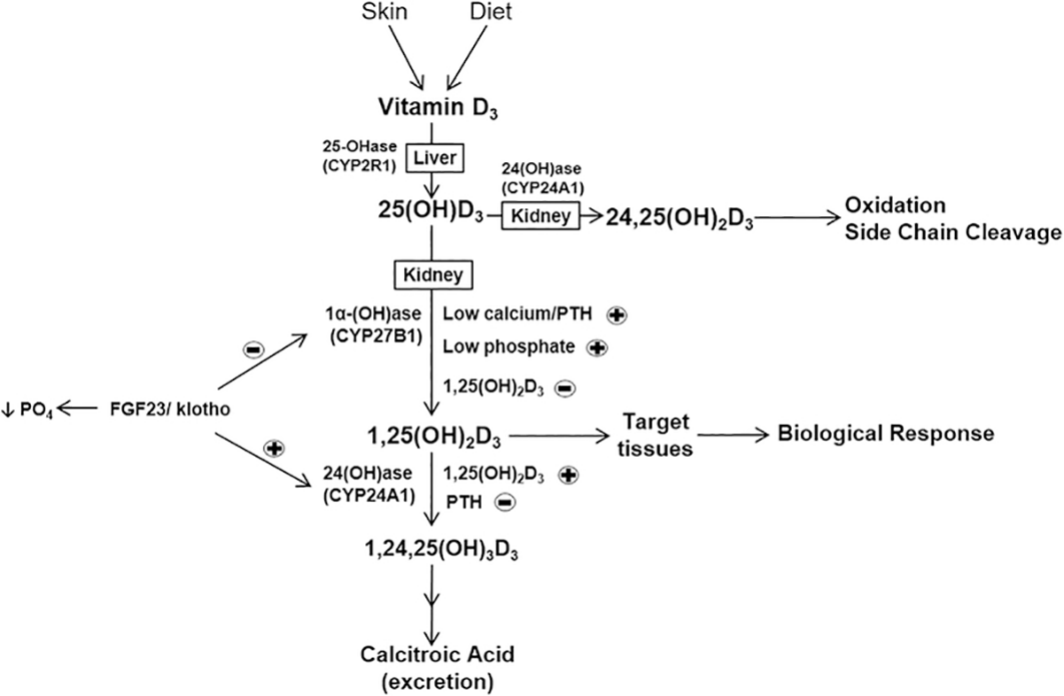
Metabolic pathway of vitamin D and its key regulatory factors. PTH and low calcium/phosphate upregulate CYP27B1 (synthesis), while 1,25(OH)₂D₃ and FGF23 upregulate CYP24A1 (degradation). The FGF23/Klotho system inhibits 1α-hydroxylase and reduces serum phosphate (33).

The positive and negative feedback mechanisms of both PTH and FGF23 regulate the dynamic balance of calcium-phosphorus metabolism. At the molecular mechanism level, these regulatory factors exert their effects through specific genomic regulatory modules; PTH activates CYP27B1 transcription through the cAMP-PKA-CREB signaling pathway, while FGF23 inhibits CYP27B1 and activates CYP24A1 by activating the MAPK signaling pathway through the FGF receptor-Klotho complex (31). The expression of CYP24A1 is transcriptionally activated by the 1,25(OH)_2_D_3_-VDR complex, forming a classic negative feedback regulatory loop that ensures precise regulation of vitamin D metabolism. This complete metabolic network not only ensures the timely activation of vitamin D biological activity but also ensures the prompt clearance of its metabolites, maintaining the dynamic balance of calcium-phosphorus metabolism in the body (32) (**Figure 3**).

The pathogenesis of clinical diseases is closely related to the aforementioned regulatory factors. In patients with chronic kidney disease, due to renal function impairment, CYP27B1 activity may not be effectively activated even when PTH levels are elevated, leading to 1,25(OH)_2_D_3_ deficiency (34). In FGF23-related diseases such as X-linked hypophosphatemia, overexpressed FGF23 continuously inhibits the production of 1,25(OH)_2_D_3_, causing severe bone mineralization disorders (35). Therefore, understanding the interactions of these regulatory mechanisms provides a theoretical basis for individualized vitamin D supplementation and novel therapeutic approaches such as FGF23 antagonists for bone mineral metabolism disorders.

#### 3.2.2 Potential connection between 1,25(OH)_2_D and 24,25(OH)_2_D

Traditional thinking considers 24,25(OH)_2_D_3_ merely as an inactive excretory product in the catabolic process of 1,25(OH)_2_D_3_, regarded as “waste” of vitamin D metabolism (36). However, recent studies are challenging this view, revealing that 24,25(OH)_2_D_3_ may possess independent biological functions rather than being merely an end-product of metabolism (37). Particularly in certain pathological states, such as in chronic kidney disease (CKD)-related mineral and bone metabolism abnormalities, researchers have observed that when 1,25(OH)_2_D_3_ levels in the body significantly decrease, the concentration of 24,25(OH)_2_D_3_ often shows a synchronous decline (30). This coordinated biochemical change pattern suggests that in the metabolic pathway of vitamin D, there may exist a more complex physiological connection between 24,25(OH)_2_D_3_ and 1,25(OH)_2_D_3_ than previously understood; they may jointly participate in the fine regulation of certain key enzyme activities or play some synergistic role in maintaining calcium-phosphorus homeostasis that has not been fully elucidated, rather than a simple “synthesis and degradation relationship. Clinical studies in chronic kidney disease patients have found that as renal function deteriorates, not only does the synthesis capacity of 1,25(OH)_2_D_3_ decline, but 24,25(OH)_2_D_3_ levels also significantly decrease (31). End-stage renal disease patients exhibit severe dual deficiency of both 1,25(OH)_2_D_3_ and 24,25(OH)_2_D_3_, accompanied by severe bone mineral metabolism disorders, secondary hyperparathyroidism, and hyperphosphatemia (28). Clinical studies in patients with osteogenesis imperfecta show that patients not only have 1,25(OH)_2_D_3_ deficiency but also significantly reduced 24,25(OH)_2_D_3_ concentrations; more importantly, targeted supplementation of 24,25(OH)_2_D_3_ can significantly increase bone density and improve bone quality, an effect independent of 1,25(OH)_2_D_3_ action (35). Recent animal experiments have confirmed that 24,25(OH)_2_D_3_ regulates chondrocyte differentiation and skeletal development through unique receptor pathways (38).

Based on the above research findings, clinical practice has begun to use the ratio of 1,25(OH)_2_D_3_ to 24,25(OH)_2_D_3_ (vitamin D ratio, VDR) as a new indicator for assessing vitamin D metabolic status (37). VDR can more accurately reflect CYP24A1 enzyme activity and the overall balance of vitamin D metabolism; when VDR is abnormally elevated, it often indicates impaired 24,25(OH)_2_D_3_ synthesis or accelerated clearance (39). Therefore, it is now believed that 24,25(OH)_2_D_3_ and 1,25(OH)_2_D_3_ synergistically maintain calcium-phosphorus homeostasis and bone health.

## 4 Studies on the correlation between vitamin D metabolism and diseases

### 4.1 Vitamin D-dependent rickets type 1A

Vitamin D-dependent rickets type 1A (VDDR-1A) is an extremely rare autosomal recessive genetic disorder that occurs in patients with autosomal recessive inheritance caused by CYP27B1 gene mutations, typically with onset in infancy. The pathogenic mechanism involves loss-of-function mutations in the CYP27B1 gene leading to severely impaired 1α-hydroxylase activity, rendering it unable to generate the biologically active form of 1,25(OH)_2_D_3_ (40). The clinical phenotype of VDDR-1A is highly characteristic, mainly manifesting as skeletal system abnormalities and neuromuscular symptoms caused by calcium-phosphorus metabolism disorders; affected children often present with acute hypocalcemic symptoms such as laryngospasm, tetany, and epileptiform seizures (35). Laboratory examination reveals a characteristic biochemical profile: serum calcium ion concentration is significantly decreased, blood phosphorus levels are usually normal or mildly decreased, alkaline phosphatase is markedly elevated, PTH levels are compensatorily increased; most importantly, the diagnostic marker 25(OH)D_3_ levels are normal or even adequate, but 1,25(OH)_2_D_3_ concentration is severely decreased or undetectable, a biochemical feature that clearly distinguishes it from nutritional vitamin D deficiency rickets (41). The treatment strategy for VDDR-1A is based on direct supplementation with active vitamin D analogs, thereby bypassing the 1α-hydroxylase defect; calcitriol is the drug of choice, with a typical starting dose of 0.25-0.5 μg/day, with individualized adjustments based on blood calcium, phosphorus, and PTH levels (42).

### 4.2 Chronic kidney disease

The kidney is a key organ for vitamin D activation and metabolism, playing a crucial role in vitamin D metabolism. As renal function declines, renal 1α-hydroxylase activity progressively decreases, leading to insufficient production of the active vitamin D metabolite 1,25(OH)_2_D_3_ (calcitriol), often accompanied by 25(OH)D_3_ deficiency. These alterations in vitamin D metabolism have been mechanistically established to contribute to the pathogenesis of secondary hyperparathyroidism (SHPT) through well-characterized pathways: reduced calcitriol levels diminish intestinal calcium absorption and impair negative feedback regulation of PTH secretion, while 25(OH)D_3_ deficiency further exacerbates parathyroid gland dysfunction (43). During CKD progression, complex vitamin D metabolic abnormalities occur, involving not only 25(OH)D_3_ and 1,25(OH)_2_D_3_ but also multiple degradation products (44). In CKD patients, the 24-hydroxylated product 24,25(OH)_2_D_3_ and its ratio to 25(OH)D_3_ (24,25(OH)_2_D_3_:25(OH)D_3_) precisely decrease with declining renal function. Notably, the ratio of 1,24,25(OH)_3_D_3_ (a downstream metabolite of 1,25(OH)_2_D_3_) to 1,25(OH)_2_D_3_ (1,24,25(OH)_3_D_3_:1,25(OH)_2_D_3_) shows an increasing trend in CKD. This indicates that CKD patients have unique abnormal vitamin D metabolic characteristics, manifested as downregulation of the 24-hydroxylation pathway and accelerated abnormal degradation of 1,25(OH)_2_D_3_.

An important complication during CKD progression is chronic kidney disease-mineral and bone disorder (CKD-MBD) (45). Clinically, CKD-MBD is mainly characterized by skeletal abnormalities (renal osteodystrophy), vascular or soft tissue calcification, and significantly increases the risk of cardiovascular events and all-cause mortality in patients. Laboratory examination shows characteristic changes: serum calcium levels may be decreased or normal, while blood phosphorus levels typically gradually increase with worsening renal function. Regarding vitamin D metabolite detection, serum 1,25(OH)_2_D_3_ levels may decline in the early stages of disease, while 25(OH)D_3_ levels are generally low. Therefore, regular combined detection of 25(OH)D_3_, 1,25(OH)_2_D_3_, and their metabolites (especially 24,25(OH)_2_D_3_ and vitamin D metabolite ratios) has indispensable value for assessing vitamin D status in CKD patients, differentiating the etiology of SHPT, and guiding precise treatment (44). Supplementation strategies for CKD patients have reached a new international consensus. The 2025 European Consensus Statement (Evenepoel et al., 2025) emphasizes that maintaining adequate substrate levels of 25(OH)D is a prerequisite for ensuring local bone-derived production of active vitamin D, offering superior benefits for maintaining intracellular metabolic homeostasis compared to the use of active analogs alone (45). For active SHPT, treatment with active vitamin D (such as calcitriol) or its analogs (such as paricalcitol) is required to directly supplement the active form, bypassing the functional defect of renal 1α-hydroxylase (45). During treatment, close monitoring of blood calcium, phosphorus, and intact parathyroid hormone levels is essential to achieve individualized dosing and avoid treatment risks such as hypercalcemia.

### 4.3 Tumor-induced osteomalacia

Tumor-induced osteomalacia (TIO) is a rare paraneoplastic syndrome; the pathogenic mechanism is generally believed to be abnormal secretion of FGF23 by benign mesenchymal tissue-derived phosphaturic mesenchymal tumors (46). Clinical manifestations include progressive, non-specific bone pain and muscle weakness, which may be accompanied by pathological fractures and limited mobility; these non-specific symptoms often lead to diagnostic delays, with an average delay time of several years (46). In the pathophysiology of TIO, the potent inhibition of CYP27B1 by FGF23 coupled with its induction of CYP24A1 creates a "double-hit" effect on vitamin D metabolism. Florenzano et al. (2025) suggest that this imbalance results in highly diagnostic metabolite patterns, providing a theoretical foundation for restoring vitamin D homeostasis via targeted FGF23 antibody therapy (47). Therefore, laboratory examination shows significantly decreased serum phosphorus levels, decreased tubular phosphorus reabsorption rate, elevated Alkaline Phosphatase (ALP) levels, serum 1,25(OH)_2_D_3_ levels that are typically decreased or inappropriately normal, while 25(OH)D_3_ levels are generally normal. Of particular importance is the detection of vitamin D metabolites including 1,25(OH)_2_D_3_ and its major degradation product 24,25(OH)_2_D_3_, as well as calculating the ratio between the two (24,25(OH)_2_D_3_:1,25(OH)_2_D_3_).In TIO, due to direct inhibition of 1α-hydroxylase by excessive FGF23, 1,25(OH)_2_D_3_ levels are suppressed while the 24-hydroxylation pathway compensatorily increases, resulting in a significantly elevated ratio. This metabolic characteristic is distinctly different from the metabolic patterns of other hypophosphatemic bone diseases (such as vitamin D-dependent rickets), thus having critical differential diagnostic value for distinguishing TIO from other diseases. Clinical treatment still prioritizes complete surgical resection of the causative tumor as the preferred method; postoperatively, patient blood phosphorus and FGF23 levels can often rapidly return to normal (48). For patients in whom the tumor cannot be localized or surgically removed, treatment mainly relies on oral phosphate preparations combined with active vitamin D (such as calcitriol) to correct hypophosphatemia and improve bone disease symptoms; close monitoring of blood calcium, phosphorus, and renal function is necessary during treatment to prevent treatment-related complications.

### 4.4 Granulomatous diseases

Granulomatous diseases, such as sarcoidosis, have a pathogenic mechanism related to abnormal immune responses of the body to specific antigens, characterized by well-demarcated nodular lesions composed of macrophages and their evolved epithelioid cells and multinucleated giant cells (49). In sarcoidosis, the granulomatous tissue itself can be unrestrained by normal regulatory mechanisms, ectopically overexpressing CYP27B1, thereby excessively converting 25(OH)D_3_ to the biologically active 1,25(OH)_2_D_3_, leading to increased intestinal calcium absorption and causing hypercalcemia (50). Clinical manifestations include, in addition to primary symptoms, hypercalcemia, related neuromuscular symptoms (such as fatigue and muscle weakness), and renal manifestations (such as polyuria and nephrocalcinosis) (51). Laboratory examination reveals elevated serum calcium ion concentration, PTH levels that are typically suppressed by feedback and decreased or at the lower limit of normal, inappropriately elevated serum 1,25(OH)_2_D_3_ levels, while 25(OH)D_3_ levels are usually normal (50). Therefore, combined detection of 1,25(OH)_2_D_3_ and PTH has critical diagnostic value for differentiating PTH-independent hypercalcemia caused by granulomatous diseases from hypercalcemia due to other causes (such as primary hyperparathyroidism or malignancy).In terms of treatment, for patients with granulomatous diseases accompanied by symptomatic hypercalcemia, glucocorticoids (such as prednisone) are the first-line therapy of choice, effectively reducing the abnormal production of 1,25(OH)_2_D_3_ and blood calcium levels by inhibiting macrophage 1α-hydroxylase activity (50).

### 4.5 Hypercalcemia caused by CYP24A1 deficiency

CYP24A1 deficiency-related hypercalcemia is a rare disease caused by loss-of-function mutations in the CYP24A1 gene, which encodes the key 24-hydroxylase of the vitamin D endocrine system responsible for degrading active vitamin D (1,25(OH)_2_D_3_) and its precursor 25(OH)D_3_ to maintain metabolic balance; mutations lead to loss of enzyme activity, causing accumulation of both vitamin D metabolites in the body and synergistic activation of VDR, ultimately triggering hypercalcemia (51). Both children and adults can be affected, with typical manifestations of hypercalcemia and hypercalciuria, often accompanied by nephrolithiasis and nephrocalcinosis; the condition can significantly worsen during pregnancy and lactation, and there is a gene-dose effect-–biallelic variant carriers have more severe nephrolithiasis and hypercalciuria, while monoallelic variant carriers have milder symptoms (52). Vitamin D metabolite detection provides critical diagnostic value: elevated serum 1,25(OH)_2_D_3_ levels, extremely low or undetecTable 24,25(OH)_2_D_3_ levels, significantly elevated 25(OH)D_3_/24,25(OH)_2_D_3_ ratio, accompanied by suppressed PTH levels. Treatment needs to target the vitamin D synthesis pathway. First, restricting vitamin D intake and sun exposure is a fundamental measure. Second, azole drugs such as fluconazole and ketoconazole can reduce the production of active vitamin D metabolites by inhibiting the cytochrome P450 enzyme system. In addition, calcium-sensing receptor agonists (such as cinacalcet) can reduce intestinal calcium absorption to maintain normal blood calcium; clinical practice requires dynamic adjustment of the regimen based on vitamin D metabolite and blood calcium levels (53).

## 5 Conclusion and outlook

In summary, vitamin D metabolism and regulation are closely related to the occurrence and development of numerous diseases. A series of metabolic processes including hepatic 25-hydroxylation, renal 1α-hydroxylation, and 24-hydroxylation in target organs are precisely regulated; abnormalities in regulatory factors such as PTH and FGF23 can trigger complex cascade reactions, leading to multi-organ system diseases such as mineral and bone metabolism disorders and kidney diseases. Therefore, in-depth understanding of vitamin D metabolic processes and regulatory mechanisms, starting from disease pathogenesis, provides scientific basis for individualized and precise clinical treatment. However, current research on the relationship between vitamin D metabolism and diseases remains primarily at the theoretical stage and has not been fully applied to clinical practice, mainly for two reasons: First, understanding of disease pathogenesis is not comprehensive enough. The pathogenic mechanisms of many diseases related to abnormal vitamin D metabolism are still being continuously deepened in understanding, and the fine regulatory mechanisms of local activation, metabolism, and inactivation of vitamin D in different organs and tissues are not yet fully understood (2). Second, biomarker detection of vitamin D metabolic processes is not comprehensive enough (20). The vast majority of clinically detected vitamin D-related indicators are still limited to 25(OH)D_3_, which is far from sufficient to comprehensively assess metabolic dynamics; the clinical application of key markers such as 1,25(OH)_2_D_3_, 24,25(OH)_2_D_3_, and metabolite ratios remains uncommon, and most scholars’ research on the diagnostic value and therapeutic guidance role of these markers in specific diseases is still not sufficiently in-depth.

Therefore, in the future, standardized protocols including multi-parameter combined detection should be established, promoting the clinical application of high-precision detection technologies such as liquid chromatography-tandem mass spectrometry, strengthening clinical translational research, and establishing disease-specific vitamin D metabolism characteristic databases to provide laboratory evidence for individualized precision diagnosis and treatment (54).
